# Health professionals’ knowledge, attitudes, and practices in snakebite management: A study from high-burden areas in the Afar Region, Ethiopia

**DOI:** 10.1371/journal.pntd.0013713

**Published:** 2025-11-20

**Authors:** Abebe M. Aga, Demise Mulugeta, Abera Motuma, Bilise Wakitole, Dassalegn Muleta, Henok Ferede, Zinash Teferi, Shambel Tadesse, Tigist Abebe, Fisseha Alemayehu, Dinkinesh Dube, Serkadis Oljira, Anberber Alemu, Gashaw Gebrewold, Fanos Tadesse Woldemariyam, Dereje Nigussie

**Affiliations:** 1 Vaccine, Diagnostics and Medical Device R&D, Armauer Hansen Research Institute, Addis Ababa, Ethiopia; 2 Malaria and other Neglected Tropical Disease Research, Ethiopian Public Health Institute, Addis Ababa, Ethiopia; Universidade Federal do Amazonas, BRAZIL

## Abstract

**Background:**

Snakebite envenoming remains a significant public health challenge, particularly in rural and resource-limited tropical regions such as sub-Saharan Africa. In Ethiopia, the Afar Region bears a disproportionate burden due to environmental exposure, pastoralist livelihoods, and limited access to timely medical care. This study aimed to assess the knowledge, attitudes, practices (KAP), and capacity gaps among healthcare professionals in selected snakebite hotspot areas of the Afar Region.

**Methods:**

A cross-sectional descriptive study was conducted in five hospitals across snakebite hotspot areas in Afar. A purposive sample of 141 healthcare professionals selected, including nurses, doctors, and other clinical staff. Data were collected using structured questionnaires addressing knowledge of envenoming, antivenom use, clinical management protocols, and facility readiness.

**Results:**

Among the healthcare professionals surveyed, nurses comprised the largest group (61%), followed by medical doctors (30.5%), with the majority of respondents (54.6%) having less than three years of professional experience. Despite their frontline role, only 1.4% of participants had received specific training related to snakebite management, while 48.9% expressed need for such training. Antivenom unavailability reported by 56% of respondents indicating high cost (17%) and frequent shortages (25.5%) as key barriers to access. Standardized clinical protocols and post-discharge follow-up practices were lacking, with 61% of care providers reporting patients did not receive any follow-up care. Snakebite cases were regularly encountered, with 36.2% of respondents indicating monthly cases and 31.2% weekly. Although antivenom was the primary first-line treatment (60.3%), the use of diagnostic methods to support case identification was not available.

**Conclusion:**

This study highlights critical deficiencies in healthcare provider training, clinical capacity, and practical experience for effective snakebite case management. Addressing these gaps requires urgent implementation of targeted training programs, development of standardized treatment protocols, and reinforcement of antivenom supply systems. Integrating snakebite management into medical and health science curricula is essential to build sustainable clinical competency and enhance patient outcomes in high-burden settings.

## Author summary

Snakebite envenoming is a major public health problem in many low-resource settings, causing significant morbidity and mortality, yet it often receives limited attention in healthcare training. This study assessed the knowledge, attitudes, and practices of healthcare professionals involved in snakebite management in high-burden areas of the Afar Region, Ethiopia. Using a cross-sectional survey, we included doctors, nurses, and laboratory personnel who play active roles in patient care, from first response to follow-up. Our findings revealed critical gaps as very few healthcare workers had received specific training on snakebite, antivenom availability was limited, and standardized management protocols were often lacking. Laboratory staff, in particular, contribute directly to patient assessment and antivenom preparation, highlighting the multidisciplinary nature of care in resource-limited settings. These results emphasize the need for targeted training programs, improved antivenom supply, and integration of snakebite management into medical education, to reduce complications and improve outcomes for affected communities.

## Introduction

Snakebite envenoming remains a global public health concern, particularly in rural and tropical regions, where healthcare systems often face significant challenges. The World Health Organization (WHO) estimates that approximately 5.4 million people worldwide are bitten by snakes, leading to 1.8 to 2.7 million cases of envenoming. Tragically, between 81,000 and 138,000 people die annually from snakebites, and roughly three times as many suffer permanent disabilities, including limb amputations [1]. The majority of these incidents occur in low and middle-income countries, where timely access to medical care and antivenom is frequently unavailable, exacerbating the disease burden.

The impact of snakebite envenoming is disproportionately high in sub-Saharan Africa, where poor healthcare infrastructure, limited access to antivenom, and delayed treatment contribute to significant morbidity and mortality. A recent study highlighted that Africa accounts for over 30% of global snakebite fatalities, with the highest burden observed in rural regions dependent on agricultural activities [2,3].

In Ethiopia, snakebite envenoming is a critical public health issue, with an estimated annual incidence rate of 16 per 100,000 people [4,5]. The puff adder is widely distributed across sub-Saharan Africa and is one of the commonest causes of clinically significant bites where it occurs. In Ethiopia; vipers (*Bitis spp*.), cobras (*Naja spp*.) and several other genera account for most envenoming reported from hotspot areas [6]. Regional data indicate that the prevalence of snakebite is particularly high in arid and semi-arid areas such as the Afar region, where the presence of venomous snakes such as *Echis carinatus* (saw-scaled viper) and *Naja nigricollis* (spitting cobra) is notable [6–8]. In the Afar Region of Ethiopia, with an incidence of approximately 150 cases per 100,000 population annually, resulting in severe complications in up to 25% of cases and a case fatality rate of 3.3% [9]. These areas are characterized by geographic remoteness, limited healthcare infrastructure, and delayed access to medical facilities, factors that contribute to poor outcomes for snakebite victims [10].

A study in Sri Lanka found that only 39% of medical officers were confident in identifying venomous bites and implementing appropriate treatment protocols [11]. Lack of standardized guidelines, fear of adverse reactions from antivenom, and absence of continuous medical education were common barriers. In sub-Saharan Africa, where antivenom availability is low and case reporting is fragmented, healthcare providers often rely on inadequate or traditional methods [12]. A study in Nigeria revealed that only 26% of healthcare workers had received any formal training in snakebite management, and misconceptions about first aid measures were widespread [13]. A study conducted in in Kenya indicate that healthcare providers lacked awareness of species-specific treatment protocols, leading to inconsistent care and delayed referrals [14]. In Uganda, the rural health facilities not only lacked antivenom but also had no standard emergency protocol for managing bites, resulting in poor outcomes [15].

In the Afar region, delayed treatment and limited access to antivenom, compounded by geographic and socio-economic factors such as pastoralist lifestyles and remote settlements, often lead to patients arriving at health facilities more than seven days after the bite [9]. Pre-hospital care for snakebite remains limited and often harmful as many victims in rural areas first seek traditional healers or use unsafe first-aid (incisions, tourniquets, suction), which delays timely treatment [9]. Prompt recognition of symptoms, accurate diagnosis, timely antivenom administration, supportive care, and adherence to evidence-based protocols improve patient outcomes. Continuous monitoring, post-discharge follow-up, culturally responsive care, and integration of snakebite management into primary healthcare systems are also essential. There is no prior KAP data for healthcare professionals in the region; however, experiences from other studies highlight the need to address knowledge gaps and strengthen snakebite management [12,16–18]. Despite global recognition of healthcare providers’ role in snakebite management, evidence from Ethiopia is limited. This study assesses the knowledge, attitudes, and practices of health workers in high-risk areas of the Afar Region, identifying capacity gaps to inform targeted training and interventions aimed at improving clinical preparedness and patient outcomes.

## Materials and methods

### Ethics statement

Ethical clearance was obtained from the Institutional Review Board (IRB) of the Ethiopian Public Health Institute. Written informed consent was obtained from all study participants prior to their interviews. Confidentiality and anonymity of participant responses were strictly maintained throughout the study, with all data securely stored and coded to protect privacy.

### Study design

A descriptive cross-sectional study was conducted from June 19, 2024 to July 12, 2024, to assess the knowledge, attitudes, practices, and capacity gaps of healthcare providers in snakebite management. The study utilized both quantitative and qualitative data to identify key challenges and gaps within the healthcare system related to snakebite care utilizing questionnaire adapted from similar previous study and pre-tested on 5 people before actual sample collection.

### Study area and setting

The Afar Region of Ethiopia presents a high-risk environment for snakebite envenoming due to its unique combination of environmental and socio-economic factors. Its semi-arid climate, sparse population, and close proximity to natural snake habitats create conditions that heighten the risk of human-snake encounters. To explore the challenges and capacity gaps in snakebite management, this study was conducted in five strategically selected healthcare facilities: Dubti, Asaita, Mohamed Akle, Gewane, and Dalifage. These hospitals serve as key referral centers for snakebite case management in the region and were chosen for their frontline role in delivering care to affected populations. Their location and function make them ideal sites for assessing the preparedness and experiences of healthcare providers in managing snakebite cases.

### Study population

The study population consisted of healthcare professionals, including doctors, nurses, health officers, and paramedics, who were directly involved in managing snakebite cases in selected facilities. The inclusion criteria required participants to be actively engaged in diagnosing, treating, or referring snakebite cases, employed at one of the selected facilities, and willing to provide informed consent. The study was conducted using a proportionate random sampling method, including approximately 24% of health professionals directly involved in snakebite case management. Healthcare professionals who were unavailable during the data collection period were excluded from the study.

### Data collection procedures

A purposive sampling method was employed to select 141 healthcare professionals from a total pool of 582 staff members across five healthcare facilities. The decision to sample approximately 24% of the workforce was guided by the need to balance feasibility with sufficient representation across different professional roles, levels of clinical experience, and facility contexts. While not designed for statistical generalization, this approach aligns with established practices in qualitative and descriptive cross-sectional studies, where capturing variability and depth of insights is prioritized over random probability sampling. By strategically including nurses, doctors, and health officers from both referral and primary-level facilities, the study ensured that volunteer’s respondent reflected the diversity of health workers directly involved in snakebite case management in high-burden areas. Only volunteers who provided informed consent were included in the study.

Data collection was conducted by independent staffs using a structured, interviewer-administered questionnaire, designed to comprehensively assess the key components of snakebite management. The instrument was organized into four sections aligned with the study objectives. The knowledge assessment section evaluated participants’ understanding of clinical signs of envenoming, appropriate use of antivenom, and adherence to management protocols. The attitudes assessment section explored healthcare providers’ perceptions of snakebite management challenges and their readiness to improve care. The practices assessment section examined routine clinical responses to snakebite cases and barriers to effective treatment, including resource constraints and treatment delays. Lastly, the capacity gaps assessment reviewed the availability of essential resources such as antivenom and diagnostics as well as prior training in snakebite management. This structured and targeted methodology enabled a systematic evaluation of critical factors influencing snakebite care in the study setting.

### Data analysis

Data were carefully curated, entered, and managed using SPSS version 20. Descriptive statistics were employed to summarize participant demographics and KAP scores. Further analyses were conducted to identify factors associated with inadequate knowledge, attitudes, practices, capacity gaps, and chi-square test were used to see association between variables. We considered a p-value of <0.05 as statistically significant for all analyses.

## Result

**Fig 1:**
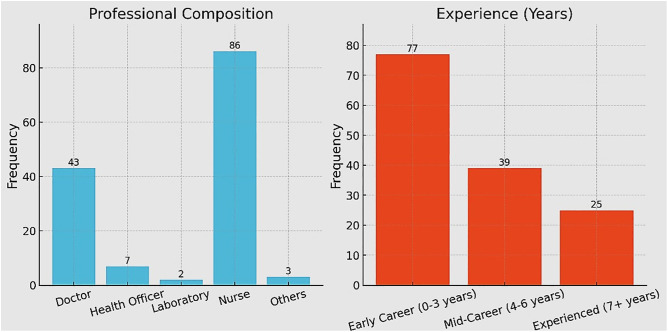
Professional distribution and experience of healthcare providers involved in snakebite management in the study hospitals.

The majority of healthcare providers managing snakebite cases were nurses (61%), followed by medical doctors (30.5%) (Fig 1). Study participants with 0–3 years of professional experience comprised 54.6% of the total, while 30.5% had 4–6 years of experience. A smaller proportion, 14.2%, had 7 or more years of experience. The three professionals under others category were diploma-level health workers.

Among the healthcare providers, 70.3% reported using clinical signs as the primary approach for snakebite case screening, which includes bite, fangs, pain or swelling to identify the cases. The other methods (29.1%) account the second and represented by gathering history of bite from the patients (Table 1). For first-line treatment, antivenom was reported by 60.3% of respondents, while 9.9% indicated pain management, and 29.8% reported using other treatments such as antibiotics and tetanus antitoxin (TAT). Regarding the frequency of snakebite case estimation, 14.9% estimated cases daily, 31.2% weekly, 36.2% monthly, and 17.7% rarely.

**Table 1:** Case screening approaches, first-line treatment practices, and estimated frequency of snakebite case presentations among healthcare professionals.

Variables	Categories	Frequency	Percentage
Case screening approaches	Clinical	100	70.3%
	Others	41	29.1%
	Total	141	100%
First-line treatment	Antivenom	85	60.3%
	Pain Management	14	9.9%
	Others (antibiotics, Tetanus Antitoxin)	42	29.8%
Case occurrence	Daily	21	14.9%
	Weekly	44	31.2%
	Monthly	51	36.2%
	Rarely	25	17.7%
	Total	141	100%

Regarding antivenom availability, 56.0% of respondents reported it was not available, 43.3% reported availability, and 0.7% indicated that it was sometimes available (Table 2). In terms of reported challenges in snakebite case management, 25.5% cited antivenom shortage, 17.0% identified high antivenom cost, and 8.5% mentioned financial burden. Additional concerns included gaps in professional knowledge and training (5.7%), difficulty in diagnosing and managing complications (5.0%), unavailability of services or delayed patient presentation (2.8%), limited community awareness and engagement (1.4%), and lack of policy-level prioritization for snakebite care (0.7%).The category Others (35.5%) encompassed a range of barriers, including poor transport and referral systems, shortage of essential supportive care supplies, limited skilled personnel for clinical diagnosis, high workload, and reliance on traditional healers.

**Table 2:** Antivenom availability and reported challenges in snakebite case management among healthcare professionals in Afar Region, Ethiopia.

Category	Response	Frequency	Percentage
Antivenom availability	No	79	56.0%
	Yes	61	43.3%
	Occasionally	1	0.7%
	Total	141	100%
Challenges	Antivenom shortage	36	25.5%
	Financial burden	12	8.5%
	Antivenom cost	24	17.0%
	Knowledge gaps	8	5.7%
	Delay in treatment	1	0.7%
	Lack of community awareness	1	0.7%
	Managing complications	6	4.3%
	Unavailability of service	3	2.1%
	Others	50	35.5%
	Total	141	100%

From 141 healthcare providers surveyed, only 2 (1.4%) reported having received specific training on snakebite management, while 138 (97.9%) indicated they had not received such training. One respondent (0.7%) was unsure or did not provide a clear response (Table 3). When asked whether specific training on snakebite management is required, 112 participants (79.4%) responded “Yes,” indicating a perceived need for such training. The remaining 29 respondents (20.6%) stated that specific training was not required. In terms of confidence in managing snakebite cases, 57 participants (40.4%) reported feeling confident in their ability to manage such cases, while 84 (59.6%) indicated that they were not confident.

**Table 3:** Training status, training needs, and confidence levels in snakebite management among healthcare professionals.

Category	Response	Frequency	Percentage
Specific training received	Yes	2	1.4%
	No	138	97.9%
	Unknown	1	0.7%
	Total	141	100%
Specific training required	Yes	112	79.4%
	No	29	20.6%
	Toal	141	100%
Confident on snakebite management	Yes	57	40.4%
	No	84	59.6%
	Total	141	100%

Regarding the estimated severity of snakebite cases, 37 respondents (26.2%) reported low severity, with local pain and swelling at the bite site only, while 45 (31.9%) estimated moderate severity (11–30%) (Table 4). High severity with local swelling extending beyond the bite site, but not involving whole limb (31–60%) was indicated by 31 respondents (22.0%), and very high severity indicated by extensive local swelling, tissue necrosis, or compartment syndrome (>60%) by 5 respondents (3.6%). Additionally, 3 participants (2.1%) reported the severity as unknown or unspecified, and 20 (14.2%) selected “Other” as their response which indicate response not fit the defined severity ranges, and fatality varies by snake type and time to treatment. In terms of post-discharge follow-up practices required to record delayed complications, monitor systemic effects and assess treatment outcomes, 86 respondents (61.0%) reported that no follow-up was conducted after snakebite treatment, while 52 (36.9%) indicated that follow-up was performed. A small number (3 respondents, 2.1%) reported the occurrence of complications following discharge. Based on Chi-square test, there was a statistically significant difference in the distribution of perceived snakebite severity among respondents (p < 0.001).

**Table 4:** Post-discharge follow-up practices and estimates of snakebite severity.

Category	Estimated %	Frequency	Percentage	P-value
**Severity estimate**	Low Severity (0–10%)	37	26.2%	*0.009*
	Moderate Severity (11–30%)	45	31.9%	
	High Severity (31–60%)	31	21.9%	
	Very High Severity (>60%)	5	3.6%	
	Unknown/Unspecified	3	2.1%	
	Other	20	14.2%	
	Total	141	100%	
**Post-discharge follow-up**	No	86	61.0%	
	Yes	52	36.9%	
	Complication	3	2.1%	
	Total	141	100%	

Of the 141 healthcare professionals surveyed, nurses constituted the largest group (n = 86), with 47 (54.7%) reporting awareness of snakebite management (Table 5). Among doctors (n = 43), only 5 (11.6%) indicated awareness, while the majority (n = 38; 88.4%) did not indicate. Among health officers (n = 7), 3 reported awareness and 4 did not, whereas all laboratory professionals (n = 2) lacked awareness of detail snakebite treatment approaches. In the ‘other’ group, which included diploma-level practitioners and midwives (n = 3), 2 reported awareness. In general, 57 respondents (40.4%) reported awareness of snakebite management, while 84 (59.6%) lacking, revealing a significant knowledge gap particularly among doctors. As tested by Chi-square, there was a statistically significant association between profession and snakebite awareness (*p < 0.001*). Regarding training, 97.9% of participants reported having received no specific training in snakebite management. Among nurses, only 2 (2.3%) had received training, while doctors, health officers, laboratory professionals, and others had no such training. Unlike awareness, the chi-square test association between profession and training status was not statistically significant (*p > 0.001*), suggesting that the lack of training was consistent across all professional groups.

**Table 5:** Cross-tabulation of healthcare professionals’ snakebite awareness and training status by profession, with corresponding p-values.

	Snakebite awareness			
Profession	No	Yes	Total	*P-value*
Doctor	38	5	43	*0.000*
Health Officer	4	3	7	
Laboratory	2	0	2	
Nurse	39	47	86	
Other	1	2	3	
Total	84	57	141	
	**Specific training received**			
Profession	**Yes**	**No**	**Total**	***P-value***
Doctor	0	43	43	*0.982*
Health officers	0	7	7	
Laboratory	0	2	2	
Nurse	2	84	86	
Other	0	3	3	
Total	2	139	141	

## Discussion

Snakebite envenoming remains a neglected tropical disease, especially in rural and resource-limited settings where health systems face major constraints. Our study, conducted in high-burden areas of Ethiopia’s Afar Region, assessed the knowledge, attitudes, and practices of healthcare providers and revealed substantial gaps in training, resource access, and clinical confidence. These challenges reflects observations from other endemic regions and underscore the urgent need for standardized training and system-level support to strengthen snakebite management.

Among 141, 56% of the respondents reported that antivenom was not available at their facilities, while less than half had consistent access. This finding is consistent with reports from Nigeria, where antivenom shortages have been associated with increased mortality and treatment delays [3]. Similar challenges have been documented in Uganda and India, where limited supply and high costs continue to impede effective snakebite care [15,19]. In the current study, antivenom shortages were identified as the most pressing challenge by a considerable number of participants, followed by high treatment costs and broader financial constraints. These limitations significantly hinder the capacity of healthcare providers to administer timely and effective treatment.

Nearly all participants reported having received no specific training in snakebite management. Despite this, the majority expressed a clear need for such training, while less than half felt confident in managing snakebite cases. These findings align with studies conducted in Kenya and India, which demonstrated that inadequate training among healthcare providers compromises the appropriate use of antivenom and adherence to emergency care protocols [14,20]. The WHO similarly emphasized the importance of clinical competency in snakebite management as a key determinant of improved patient outcomes, reinforcing the need for targeted and context-specific training interventions.

Clinical screening methods for snakebite cases were primarily based on general clinical signs, while a portion of respondents reported using other non-specific approaches as supportive care that relieves symptoms by giving painkillers for snakebite without administering antivenom. Antivenom was commonly identified as the first-line treatment; however, a significant number of healthcare providers indicated the use of antibiotics or tetanus antitoxin, reflecting inconsistencies in treatment practices. Additionally, few facilities reported conducting any form of post-discharge follow-up, and only a small number noted documented complications. These findings highlight the absence of standardized treatment and follow-up protocols, which may contribute to missed complications and suboptimal recovery outcomes.

Inconsistent classification of complication severity may lead to misjudgment of clinical risk and influence treatment decisions, including the timing and dosage of antivenom. Such variability can result in delayed or inappropriate interventions, increasing patient morbidity and length of hospital stay. This inconsistency likely arises from the lack of uniform guidelines and limited clinical exposure, particularly among early-career healthcare professionals. Implementing standardized severity grading is therefore essential to support evidence-based management and ensure consistent patient care across facilities. Globally, similar KAP assessments highlight consistent trends revealing gaps in community awareness, diagnostic skills, and antivenom use, mirroring the findings of this study [3,21]. A study in India reported reliance on traditional remedies and delays in presentation due to poor knowledge and lack of standardized care pathways [22]. These regional studies underscore the widespread nature of the issues identified in our context and reinforce the need for coordinated global efforts to improve snakebite management.

In this study, several critical gaps were identified revealing systemic weaknesses in snakebite management among healthcare providers. First, the limited availability and high cost of antivenom emerged as major barriers to effective treatment. Many facilities reported frequent stockouts and unaffordable pricing, making it difficult to deliver life-saving care promptly. Secondly, there was a substantial training deficit, with nearly all respondents lacking formal instruction in snakebite management. This lack of training undermines healthcare workers’ confidence and ability to follow proper treatment protocols. Third, inconsistencies in clinical practice were evident, particularly in how providers assess symptoms, select treatment options, and follow up with patients. The use of non-standardized screening methods and reliance on antibiotics or supportive care in place of antivenom points to the absence of unified clinical guidelines. Additionally, post-discharge care was found to be largely inadequate, with very few facilities conducting follow-ups to monitor complications or recovery, indicating a fragmented approach to long-term patient management. The statistically significant variation in perceived snakebite severity among healthcare providers indicates inconsistent clinical experience or awareness, which may affect triage and treatment decisions. In contrast, the lack of significant association between profession and specific training received suggests that training gaps are widespread and not profession-specific, underscoring the need for universal capacity-building initiatives.

Improving pre-hospital snakebite care required with community education on safe first-aid, discouraging harmful practices, and strengthening referral systems. Frontline healthcare providers should receive in-service training on recognition, severity grading, antivenom use, and emergency response, supported by simplified algorithms. Ensuring equitable access to affordable antivenom through stronger supply chains and subsidies is critical, alongside public awareness campaigns on prevention and timely care-seeking. Longer-term priorities include developing national treatment guidelines, investing in rural health infrastructure and integrating snakebite management into medical curricula is important. Furthermore, conducting operational and epidemiological research on local snake species and envenoming patterns will support context-specific clinical approaches. Finally, advocacy for increased policy attention and funding should be prioritized to sustain progress and integrate snakebite management into broader health system strengthening efforts.

## Conclusion

This study provides important insights into the persistent challenges of snakebite management in resource-limited settings. Antivenom shortages and high costs were identified as major barriers, limiting timely access to effective treatment in many facilities. In addition, the absence of structured training and standardized protocols has left many healthcare providers with low confidence in diagnosis and case management, while post-discharge follow-up remains inconsistent, reducing opportunities to detect and manage complications. These findings underscore the urgent need for a coordinated response to strengthen health system capacity for snakebite care. Priority actions should include securing reliable antivenom supply chains, ensuring affordability, and incorporating structured snakebite management training into healthcare curricula and in-service programs. Community awareness initiatives are also essential to promote early care-seeking and reduce delays. In the longer term, the development of national guidelines, improved rural infrastructure, and locally tailored research are critical to reducing morbidity and mortality from snakebite. We call for government prioritization of snakebite envenoming and its integration into national emergency response frameworks to strengthen preparedness and reduce preventable deaths.

### Study limitations

A limitation of this study is that some responses were non-specific or qualitative, potentially reducing the precision of severity and challenge assessments. Additionally, the findings may be influenced by representativeness issues, recall bias, and limited generalizability.

## Supporting information

S1 File Health professionals KAP questionnaire. This file contains the original questionnaire used in the study to assess the knowledge, attitudes, and practices of healthcare professionals involved in snakebite management as requested by reviewer.
(DOCX)
