# Supplementary material for: Health professionals’ knowledge, attitudes, and practices in snakebite management: A study from high-burden areas in the Afar Region, Ethiopia
Source: PLoS Negl Trop Dis. 2025 Nov 20;19(11):e0013713. doi: 10.1371/journal.pntd.0013713 (PMC12633901; doi:10.1371/journal.pntd.0013713)
Supplement: S1 File — This file contains the original questionnaire used in the study to assess the knowledge, attitudes, and practices of healthcare professionals involved in snakebite management as requested by reviewer. (DOCX) [file pntd.0013713.s001.docx]

**Health professionals KAP study**

| **Category** | **Question** | **Possible options** |
| --- | --- | --- |
| **Demographics** | Participant ID |  |
|  | 1. What is your professional role? | ^Doctor/Nurse/Paramedic/Other^ |
|  | 2. How many years of experience do you have in your current role? | ^Numeric^ |
|  | 3. Which healthcare facility are you affiliated with? | ^Text^ |
| **Clinical management** | 4. How frequently do you encounter snake bite cases? | ^Daily/Weekly/Monthly/Rarely^ |
|  | 5. What are the first-line treatments you administer for a snake bite? | ^Text^ |
|  | 6. How do you assess the severity of a snakebite? | ^Text/Options (clinical signs, lab tests)^ |
|  | 7. What guidelines or protocols do you follow for snakebite management? | ^Text/Options Non/local/(national/international guidelines)^ |
| **Service & resource availability** | 8. Is anti-venom always available at your facility? | ^1. Yes/0. No^ |
|  | 9. Are there adequate facilities for monitoring and managing complications? | ^1. Yes/0. No^ |
|  | 10. Do you have access to appropriate diagnostic tools for snakebite management (lab tests, imaging)? | ^1. Yes/ 0. No^ |
| **Challenges & needs** | 11. What are the most common challenges you face when treating snakebite patients? | ^Text^ |
|  | 12. Have you undergone any specialized training for snakebite management? | ^Text^ |
| **Training & awareness** | 13. Are there any specific resources or training you feel are lacking in your facility related to snakebite management? | ^1. Yes/ 0. No, if yes describe….^ |
|  | 14. How confident are you in identifying venomous vs non-venomous snake species? | ^Scale (1-5, with 5 being most confident)^ |
|  | 15. Do you feel that there is enough awareness among the community about the immediate steps to take after a snake bite? | ^1. Yes/ 0. No^ |
| **Outcome & follow-up** | 16. What percentage of snakebite cases at your facility would you estimate result in complications or fatalities? | ^Numeric/Percentage^ |
|  | 17. Are there any follow-up procedures or check-ups recommended for snakebite victim post-discharge? | ^1. Yes/ 0. No (If yes, specify)^ |
